# The accessory renal arteries: A systematic review with meta‐analysis

**DOI:** 10.1002/ca.24255

**Published:** 2024-12-08

**Authors:** George Triantafyllou, Ioannis Paschopoulos, Andrzej Węgiel, Łukasz Olewnik, George Tsakotos, Nicol Zielinska, Maria Piagkou

**Affiliations:** ^1^ Department of Anatomy, School of Medicine, Faculty of Health Sciences National and Kapodistrian University of Athens Athens Greece; ^2^ Department of Clinical Anatomy Masovian Academy in Płock Płock Poland

**Keywords:** anatomical variation, anatomy, kidney transplantation, renal artery

## Abstract

The accessory renal arteries (ARAs) are a well‐described variant of the renal vasculature with clinical implications for radiologists, surgeons, and clinicians. The aim of the present systematic review with meta‐analysis was to estimate the pooled prevalence of ARAs, including their variant number, origin, and termination, and to highlight symmetrical and asymmetrical morphological patterns. The systematic review used four online databases in accordance with PRISMA 2020 and Evidence‐based Anatomy Workgroup guidelines. R programming software was used for the statistical meta‐analysis. A total of 111 studies were considered eligible for our initial aim. The typical renal artery (RA) anatomy (a single bilateral vessel) was identified in 78.92%; the overall ARA prevalence was estimated at a pooled prevalence of 21.10%. The estimated pooled prevalence of one, two, three, and four ARAs were 18.67%, 1.80%, 0.01%, and <0.01%. The ARAs have been the subject of extensive research owing to their clinical importance, including in kidney transplantation surgery and resistant hypertension therapy. Knowledge of the typical and variant anatomy of RAs is essential for anatomists, radiologists, surgeons, and clinicians in order to avoid misunderstanding, complications, and iatrogenic injury.

## 1 INTRODUCTION

The renal vasculature is crucial for endoscopic and transplantation surgery, interventional radiology procedures, and renal vascular interventions. Clinicians have also linked renal artery (RA) variants to RA denervation in resistant hypertension (Kasprzycki et al., 2023; Tubbs et al., 2016).

Typically, the RA is a paired vessel arising from the abdominal aorta at the level of the second or third lumbar vertebra (L2 or L3). The right RA has a longer course than the left owing to its location posterior to the inferior vena cava. The RA usually divides into two main trunks, which branch into 5‐7 arteries that enter the renal parenchyma (Lippert and Pabst, 1985; Tubbs et al., 2016). According to *Bergman's Comprehensive Encyclopedia of Anatomic Variations*, the classically‐described RA can be identified in 70%–75% of the population (Tubbs et al., 2016). The most common variant in kidney vasculature (approximate incidence 30%) is more than one RA (Tubbs et al., 2016). The numbers of accessory RAs (ARAs) can vary greatly; one to five have been reported (Coulier, 2013; Tubbs et al., 2016). An ARA can be originate from the abdominal aorta or from the main RA, and rarer origins from the common iliac artery or celiac trunk have been described. Three ARA termination patterns can be distinguished based on the area of the kidney supplied: the superior polar, the inferior polar, and the hilar arteries (Tubbs et al., 2016). Researchers have used various terms to describe the ARAs such as multiple, additional, supernumerary, aberrant, supplementary, anomalous, extra, or plural arteries (Tubbs et al., 2016).

In the current systematic review with meta‐analysis, we suggest that the term “*accessory renal artery–ARA*” should be used to denote the variation, in accordance with the Kachlík et al. (2020) proposal for variant vascular anatomy (*vas accessorium*).

Owing to their anatomical and clinical interest, the prevalence of ARAs has been widely studied in the literature. Although there have been several comprehensive reviews of the field (Gulas et al., 2016; Gulas et al., 2018; Recto et al., 2019), we conducted an evidence‐based systematic review with meta‐analysis, according to the guidelines proposed by the Evidence‐Based Anatomy Workgroup (Henry et al., 2016; Henry et al., 2017), to estimate the pooled prevalence of ARAs including their variant number, origin and termination, highlighting the pooled prevalence of symmetrical and asymmetrical patterns.

## 2 MATERIALS AND METHODS

The systematic review with meta‐analysis was conducted in accordance with the guidelines proposed by the Evidence‐based Anatomy Workgroup (Henry et al., 2016) for anatomical studies and the PRISMA 2020 (Page et al., 2021) guidelines.

Three independent reviewers performed the literature search and data extraction (GTr, IP, AW). Results were compared, and the other authors resolved potential differences. The terms “renal artery,” “variation,” “anatomical variation,” “variability,” “accessory,” “multiple,” “supernumerary,” “supplementary,” “plural,” “additional,” “double renal arteries,” “triple renal arteries,” “incidence,” “prevalence”, “anatomical study,” “imaging study” and “surgical study” were used in various combinations (Table 1) in the online databases PubMed, Google Scholar, Scopus and Web of Science, from January 2024 until April 2024. Studies reporting RA variants, especially ARAs, were considered eligible for the systematic review. There were no data or language restrictions. Case reports, animal studies, conference abstracts, letters to the editor, and studies with irrelevant, insufficient, or incomplete data were excluded. To enhance our literature search, three more resources were investigated. The eligible articles’ references, the gray literature, and significant anatomical journals (Annals of Anatomy, Clinical Anatomy, Journal of Anatomy, Anatomical Record, Surgical and Radiological Anatomy, Folia Morphology, Anatomical Science International, Anatomy and Cell Biology) were also searched hands‐on. The data were extracted using Microsoft Excel sheets before statistical analysis.

**TABLE 1:** A keyword example for the literature search.

Search number	Search term combinations
1	(renal artery) AND ((variation) OR (anatomic variation) OR (variability))
2	(renal artery) AND ((accessory) OR (additional) OR (multiple) OR (supplementary) OR (plural) OR (supernumerary))
3	((double renal arteries) OR (triple renal arteries)) AND ((incidence) OR (prevalence))
4	(renal artery) AND ((anatomical study) OR (imaging study) OR (surgical study))

The open‐source R programming language and RStudio software (version 4.3.2) with the “meta” and “metafor” packages was used for the statistical meta‐analysis. The pooled prevalence was calculated using the inverse variance and random effects models. The proportions meta‐analysis (prevalence meta‐analysis) was conducted using the Freeman‐Tukey double arcsine transformation, the DerSimonian‐Laird estimator for the between‐study variance tau^2^, and the Jackson method for confidence interval of tau^2^ and tau. Several subgroup analyses were also performed to detect variables affecting the estimated pooled prevalence. A *p*‐value less than 0.05 was considered statistically significant. Cochran's Q statistic was used to determine heterogeneity across studies, and the Higgins I^2^ statistic was used to quantify heterogeneity. Cochran's Q p‐value <0.10 was considered significant. Higgins I^2^ values between 0 and 40% were regarded as no significant heterogeneous, 30%–60% as moderate heterogeneity, 50–90% characterized as substantial heterogeneity, and 75%–100% could represent considerable heterogeneity. To evaluate a small‐study effect (smaller studies can show different effects from large ones), the DOI plot with the LFK index was generated (Furuya‐Kanamori et al., 2018).

## 3 RESULTS

**FIGURE 1:**
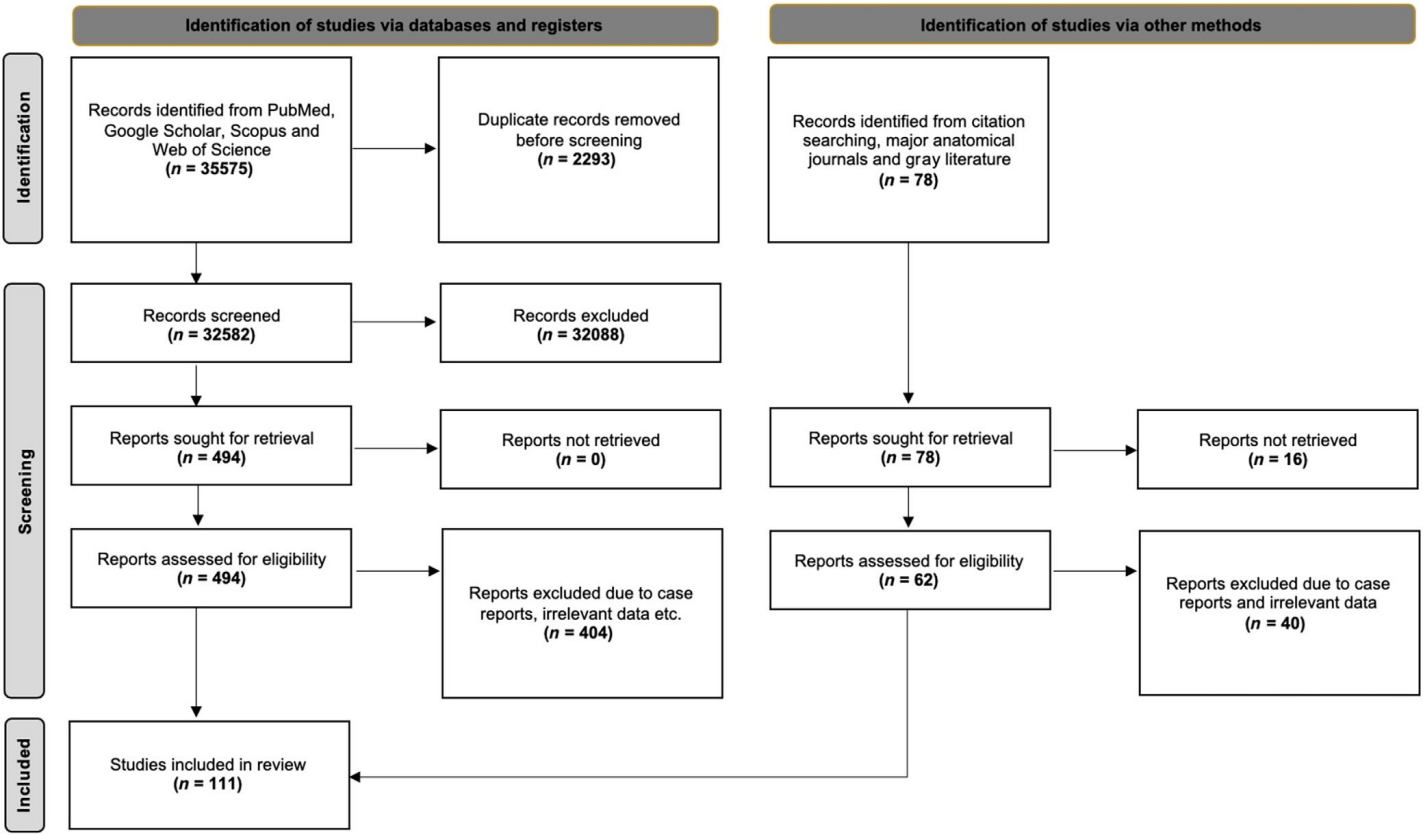
A flow chart of the search process in accordance with PRISMA 2020 guidelines (Page et al., 2021).

The database search retrieved 35,575 articles exported to Mendeley version 2.10.9 (Elsevier, London). After a check for duplicates, the titles and abstracts were assessed for eligibility. After duplicate and irrelevant papers were eliminated, 494 studies underwent full‐text retrieval and screening. Finally, 90 studies from the primary search were eligible for the systematic review. A further 21 studies were identified from our secondary search (references, gray literature, and anatomical journals hands‐on search). Thus, 111 studies were included in the current systematic review with meta‐analysis. Figure 1 is a flow diagram of the search analysis based on the PRISMA 2020 guidelines (Page et al., 2021).

One hundred and eleven studies were included, involving a total of 54,814 kidneys. Fifty‐five of the studies were imaging, 36 were cadaveric, and 20 were surgical. Twenty‐four studies had samples of fewer than 100 kidneys, and 87 had samples of more than 100. The mean number of kidneys per article was 491.2. Forty‐seven articles concerned Asian populations, 28 European and American populations, seven African populations, and two the Oceanian population. The characteristics of the eligible articles are summarized in Table 2.

**TABLE 2:** Characteristics of eligible studies including the AQUA Tool risk of bias (Henry et al., 2017).

Study, year	Population	Type of study	No. of kidneys	Age group	Risk of bias
Abdessater et al., 2022	Europe	Surgical	356	NR	High
Adachi et al., 1928	Asia	Cadaveric	338	Adults	High
Amirzargar et al., 2013	Asia	Surgical	76	Adults	High
Ankolekar & Sengupta, 2013	Asia	Cadaveric	60	NR	High
Antonopoulos et al., 2014	America	Surgical	602	Children and adults	Low
Aragão et al., 2012	America	Cadaveric	60	Fetuses (20–37 weeks)	High
Aristotle & Felicia, 2013	Asia	Cadaveric	30	Adults	High
Arpalı et al., 2020	Asia	Surgical	1350	Adults	Low
Aytac et al., 2003	Asia	Imaging (DSA)	107	Adults	Low
Bordei et al., 2004	Europe	Cadaveric	272	Adults and fetuses	High
Bouali et al., 2012	Europe	Imaging (CT)	240	Adults	Low
Bouzouita et al., 2021	Africa	Cadaveric	71	NR	High
Brewer, 1898	America	Cadaveric	302	Adults	High
Bude et al., 2003	America	Imaging (DSA)	87	Adults	Low
Budhiraja et al., 2013	Asia	Cadaveric	74	NR	High
Cases et al., 2017	Europe	Cadaveric & Imaging (CT‐64 slices)	1226	Adults	Low
Chabchoub et al., 2011	Africa	Surgical	204	Adults	High
Choi et al., 1997	Asia	Surgical	500	NR	High
Cicek et al., 2021	Asia	Imaging (CT‐64 slices)	3718	Adults	Low
Çiçekcibaşi et al., 2005	Asia	Cadaveric	180	Fetuses	Low
Çınar & Türkvatan, 2016	Asia	Imaging (CT‐64 slices)	1008	Adults	High
Costa et al., 2019	Europe	Imaging (CT and MRI)	604	NR	High
Desberg et al., 1990	America	Imaging (Doppler)	55	Adults	High
Famurewa et al., 2018	Africa	Imaging (CT)	400	Adults	Low
García‐Barrios et al., 2024	Europe	Cadaveric	16	Adults	High
Gautam et al. 2020	Asia	Imaging (CT)	34	NR	High
Gerald, 1911	Europe	Cadaveric	287	Adults	High
Geyer & Poutasse, 1962	America	Imaging	744	NR	High
Giavroglou, 1982	Europe	Imaging	1855	Adults	High
Gościcka et al., 1996	Europe	Imaging	280	Fetuses	High
Guan et al., 2013	Asia	Imaging (CT‐64slices and DSA)	214	Adults	Low
Gümüş et al., 2012	Asia	Imaging (CT‐64 slices)	1626	Children and adults	Low
Gupta & Tello, 2004	America	Imaging (MR‐1.5T)	370	Adults	Low
Gupta et al., 2011	Asia	Cadaveric	60	Adults	High
Harraz et al., 2013	Africa	Imaging (US Doppler)	731	NR	High
Hassan et al., 2017	America	Cadaveric	126	Adults	High
Hlaing et al., 2010	Asia	Cadaveric	50	NR	High
Holden et al., 2005	Asia	Imaging (CT‐16 slices)	200	NR	High
Hsu et al., 2003	America	Surgical	353	NR	High
Hung et al., 2012	Asia	Surgical and Imaging (CT)	100	Adults	Low
Jalamneh et al., 2023	Asia	Imaging (CT‐128 slices)	1100	Children and adults	High
Jamkar et al., 2017	Asia	Cadaveric	1060	Adults	High
Janschek et al., 2004	Europe	Cadaveric	238	Adults	High
Johnson et al., 2013	America	Imaging (CT‐64 slices)	604	NR	High
Kadotani et al., 2005	Asia	Surgical	340	NR	High
Kapoor et al., 2011	America	Surgical	342	NR	High
Khamanarong et al., 2004	Asia	Cadaveric	534	Children and adults	High
Kok et al., 2008	Europe	Surgical and Imaging (MRA‐1.5T and DSA)	288	Adults	Low
Kumaresan et al., 2022	Asia	Imaging (CT‐64 slices)	198	Adults	High
Laouad et al., 2012	Europe	Surgical	259	Children and adults	Low
Lauder et al., 2018	Europe and America	Imaging	2000	Adults	High
Levi, 1909	Europe	Cadaveric	1921	Adults	High
Li, Jie, et al., 2021; Li, Xia, et al., 2021	Asia	Imaging (CT and MRA‐3T)	60	Adults	High
Iijima, 1925	Asia	Cadaveric	60	Adults	High
Lloyd, 1935	America	Cadaveric	144	Adults	High
Lloyd, 1935	America	Cadaveric	162	Adults	High
Luo et al., 2019	Asia	Imaging (CT‐128 slices)	168	Children	Low
Lv et al., 2023	Asia	Imaging (CT)	198	Adults	High
Majos et al., 2018	Europe	Imaging (CT‐64 slices)	496	Adults	Low
Maleki et al., 2020	Asia	Imaging (MDCT)	258	Adults	High
Malgor et al., 2013	America	Imaging (CT‐16 or 64 slices)	238	Adults	Low
Mansur et al., 2019	Asia	Imaging (CT)	206	NR	High
Mehreen et al., 2023	Asia	Imaging (CT‐160 slices)	122	Adults	High
Meng et al., 2015	Asia	Surgical and Imaging (CT‐128 slices)	42	Adults	High
Merklin & Michels, 1958	American	Cadaveric	185	Adults	High
Mihaylova et al., 2023	Europe	Imaging (CT‐320 slices)	1122	Adults	Low
Mohammed et al., 2024	Asia	Imaging (CT‐64 slices)	800	Adults	Low
Munnusamy et al., 2016	Asia	Imaging (CT)	200	NR	High
Mustafa et al., 2016	Asia	Surgical Cadaveric and Imaging (CT‐64 slices)	549	Adults	High
Natsis et al., 2014	Europe	Cadaveric	206	Adults	Low
O'Neill et al., 2020	Europe	Imaging (MDCT‐16 or 128 slices)	320	Adults	High
Odman & Ranniger, 1968	Europe and USA	Cadaveric	280	Adults	High
Ogeng'o et al., 2010	Africa	Cadaveric	356	NR	High
Oh et al., 2003	America	Surgical and Imaging (CT)	73	Children and adults	High
Omar et al., 2021	Africa	Imaging (CT)	593	NR	High
Özkan et al., 2006	Asia	Imaging (DSA)	1697	Children and adults	Low
Palmieri et al., 2011	America	Imaging (CT‐16 slices)	200	Adults	High
Papaloucas et al., 2007	Europe	Imaging	215	Adults	High
Platt et al., 1997	America	Imaging (CT) and Surgical	307	Adults	Low
Pradhay et al., 2021	Asia	Imaging (MDCT)	100	NR	High
Ronstrom, 1947	America	Cadaveric	200	NR	High
Rubin et al., 1995	America	Imaging (CT)	24	Adults	High
Saldarriaga et al., 2008	America	Surgical	390	Children and adults	High
Sampaio, 1992	America	Cadaveric	266	Adults	High
Sanghvi et al., 2022	America and Europe	Imaging (CT or MRA)	648	Adults	Low
Sarier et al., 2020	Asia	Imaging (CT‐16 slices) and Surgical	2144	Adults	Low
Satyapal et al., 2001	Africa	Imaging and Cadaveric	440	Children and adults	High
Seldowitsch, 1909	Europe	Cadaveric	300	Adults	High
Sezer et al., 2012	Asia	Surgical	249	NR	High
Shen et al., 2021	Asia	Imaging (CT)	5908	Adults	Low
Shoja et al., 2008	America	Imaging	81	NR	High
Singh et al., 2007	America	Surgical	333	Adults	High
Soares et al., 2012	America	Cadaveric	100	NR	High
Song et al., 2020	Asia	Imaging (MDCT‐64 slices)	628	Adults	High
Sośnik & Sośnik, 2020	Europe	Cadaveric	1100	Adults	High
Stojadinovic et al., 2020	Europe	Cadaveric	110	Adults	High
Sungura, 2012	Africa	Imaging (CT)	408	Adults	High
Sykes, 1963	Europe	Cadaveric	153	Adults	High
Talović et al., 2004	Europe	Imaging	213	NR	High
Tao et al., 2013	Asia	Imaging (CT‐64 slices)	756	NR	High
Tardo et al., 2017	Australia	Imaging (CT‐128 slices) and Cadaveric	594	Adults	Low
Tarzamni et al., 2008	Asia	Imaging (CT)	234	Adults	High
Toprak et al., 2005	Asia	Imaging (CT)	20	Adults	High
Torrealba et al., 2022	America and Europe	Imaging (CT)	579	Adults	Low
Tyson et al., 2011	America	Surgical	510	Adults	High
Vecanova et al., 2023	Europe	Cadaveric	80	Adults	High
Vilhova et al., 2001	Europe	Imaging and Cadaveric	68	NR	High
Weld et al., 2005	America	Cadaveric	73	NR	High
Wozniak, 2000	Europe	Cadaveric	152	NR	High
Zağyapan et al., 2009	Asia	Imaging	300	Adults	High
Zhao et al., 2015	Asia	Imaging (MDCT)	546	Adults	Low

Abbreviations: CT, computed tomography; DSA, digital subtraction angiography; MDCT, multi‐detector computed tomography; MR, magnetic resonance; US, ultrasonography.

A single RA originating from the abdominal aorta and supplying the kidney through the hilum was considered the typical RA anatomy. It had a pooled prevalence of 78.92% (95% CI: 77.01%–80.77%); estimated bilateral presence 68.81% of pooled prevalence (95% CI: 64.71%–72.78%). The single RA was identified in 78.98% on the right side and 77.77% on the left (*p* = 0.60). Subgroup analyses showed no significant effects of geographic distribution, type of study, sample size, or sex on the RA (Tables 3, 4, and 5).

**TABLE 3:** The pooled prevalence of each renal artery (RA) parameter.

Parameters	No. of studies (kidneys)	Pooled Prevalence (%)	95% CI	I^2^ (%)	*p*‐value
Typical RA	111 (54,814)	78.92%	77.01–80.77	96.3	<0.0001
ARA Total	111 (54,814)	21.10%	19.25–23.01	96.3	<0.0001
Variation in ARA number					
One ARA	77 (33,184)	18.67%	16.72–20.69	94.9	<0.0001
Two ARAs	77 (33,184)	1.80%	1.34–2.32	86.6	<0.0001
Three ARAs	77 (33,184)	0.01%	0.00–0.04	31.5	0.0072
Four ARAs	77 (33,184)	<0.01% (6 cases)	0.00–0.00	0	1.000
Variation in ARA origin					
Abdominal aorta	17 (10,556)	88.39%	79.55–95.25	90.9	<0.0001
RA	17 (10,556)	8.34%	1.77–17.93	93.4	<0.0001
Other origins*	17 (10,556)	<0.01%	0.00–0.07	0	1.000
Variation in ARA termination					
Superior polar artery	20 (10,886)	8.02%	5.50–10.96	95.6	<0.0001
Inferior polar artery	20 (10,886)	6.69%	4.68–9.02	93.9	<0.0001
Hilar artery	20 (10,886)	8.83%	5.36–13.02	97.6	<0.0001

*Note*: A detailed overview of Higgins's I^2^ statistics for quantifying heterogeneity and Cochran's Q test (*p*‐value) for heterogeneity is provided.

Abbreviation: ARA, accessory renal artery.

* common iliac, celiac trunk, and superior mesenteric artery.

**TABLE 4:** Subgroup analysis of renal artery (RA) according to geographic distribution, type of study, and sample size.

Parameter	Typical RA	ARAs total	One ARA	Two ARAs	Three ARAs	Four ARAs
Europe (*k*=28)	78.45%	21.56%	19.12%	1.71%	0.14%	<0.01%
Asia (*k*=46)	79.54%	20.50%	18.15%	1.65%	<0.01%	<0.01%
America (*k*=28)	76.04%	23.96%	21.72%	2.35%	<0.01%	<0.01%
Africa (*k*=7)	84.80%	15.20%	13.65%	1.23%	0.09%	0.06%
Oceania (*k*=2)	83.01%	16.99%	15.60%	1.09%	0.08%	<0.01%
*p*‐value	0.2023	0.2027	0.4598	0.7149	0.2367	0.6767
Imaging (*k*=55)	79.82%	20.18%	16.77%	1.73%	0.04%	<0.01%
Cadaveric (*k*=36)	79.17%	20.84%	19.43%	1.55%	<0.01%	<0.01%
Surgical (*k*=20)	75.67%	24.43%	23.42%	2.34%	<0.01%	<0.01%
*p*‐value	0.2851	0.2680	0.0688	0.3739	0.1564	0.8525
Sample >100 kidneys (*k*=87)	79.81%	20.21%	17.56%	1.99%	0.07%	<0.01%
Sample <100 kidneys (*k*=24)	73.98%	26.02%	24.77%	1.11%	<0.01%	<0.01%
*p*‐value	0.2384	0.2404	0.2215	0.7302	0.1522	0. 8525

Abbreviation: ARA, accessory renal artery.

**TABLE 5:** The renal artery (RA) distribution is based on side, sex, and laterality.

Parameters	Typical RA	ARA total	One ARA	Two ARAs	Three ARAs	Four ARAs
Side effect						
Left	77.77%	21.02%	18.94%	2.12%	<0.01%	<0.01%
Right	78.98%	22.23%	18.60%	1.91%	<0.01%	<0.01%
*p*‐value	0.6016	0.6016	0.8753	0.7121	0.9111	0.8485
Sex effect						
Male	77.05%	22.95%	21.60%	3.08%	0.13%	<0.01%
Female	80.27%	19.73%	16.52%	1.92%	0.03%	<0.01%
*p*‐value	0.4641	0.4641	0.2210	0.4596	0.7305	0.7329
Laterality effect						
Bilateral	68.81%	‐	5.15%	0.30%	<0.01%	<0.01%

Abbreviation: ARA, accessory renal artery.

**FIGURE 2:**
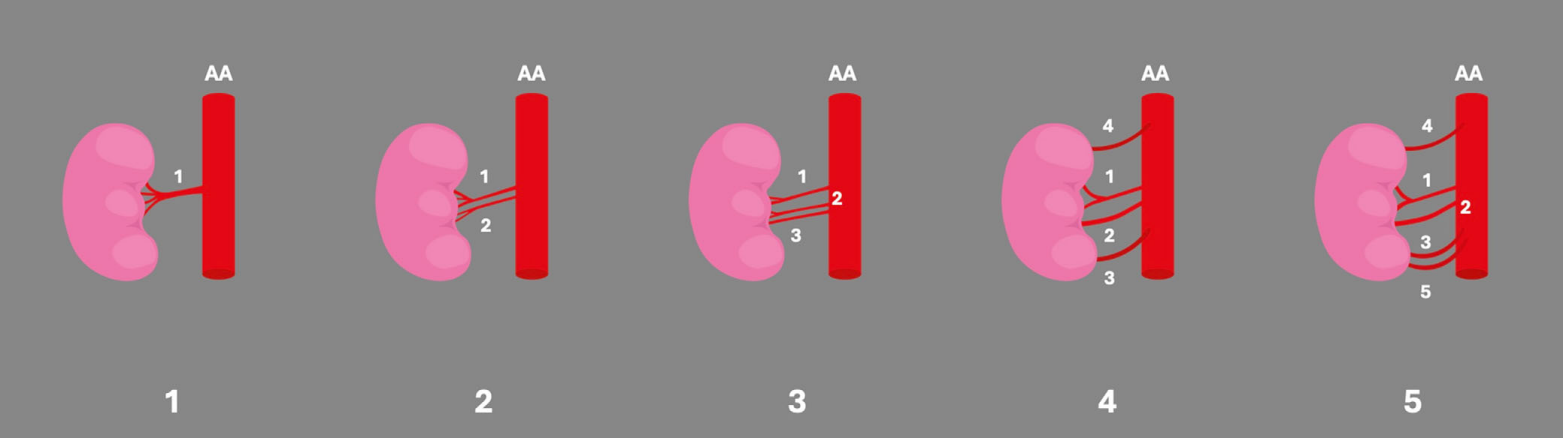
The accessory renal arteries (ARAs); (A) the typical single renal artery (RA), (B) one ARA, (C) two ARA, (D) three ARA, (E) four ARA. AA, abdominal aorta.

ARAs had an overall prevalence of 21.10% (95% CI: 19.25–23.01) (Figure 2). Subgroup analyses revealed no significant effects of geographic distribution, type of study, sample size, side, or sex on the ARA (Tables 3, 4, and 5). To detect possible nationality influence, we re‐evaluated the subgroup analysis by excluding the Oceanian subgroup studies because of their low number (*k* = 2). The geographical distribution subgroup analysis was not statistically significant (*p* = 0.1384).

One ARA (two RAs in total) was estimated with a pooled prevalence of 18.67% (95% CI: 16.72%–20.69%), bilateral in 5.15% (95% CI: 3.82%–6.64%). The pooled prevalence of the left‐sided ARA was estimated at 18.94% and the right‐sided at 18.60% (*p* = 0.88).

Two ARAs (three RAs in total) were calculated with a pooled prevalence of 1.80% (95%CI: 1.34–2.32), bilateral in 0.30% (95% CI: 0.06%–0.66%). Two ARAs on the left had a pooled prevalence of 2.12%, and on the right a pooled prevalence of 1.91% (*p* = 0.71).

Three ARAs (four RAs in total) had a pooled prevalence of 0.01% (95% CI: 0.00%–0.04%), and fewer than 0.01% were bilateral (95% CI: 0.00–0.11).

Four ARAs (five RAs) were estimated with a pooled prevalence of less than 0.01% (six cases in the literature). Subgroup analyses revealed no significant differences in these ARAs in geographic distribution, type of study, sample size, side, or sex (Tables 3, 4, and 5).

Symmetrical RA morphology (the same number of RAs bilaterally) had a pooled prevalence of 74.50% (95%CI: 71.09‐77.78%), and asymmetrical morphology (different number of RAs bilaterally) of 25.50% (95%CI: 22.22%–28.91%); the difference was significant (*p* <0.001).

**FIGURE 3:**
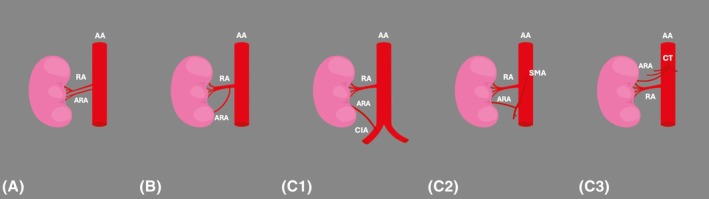
Origins of the accessory renal arteries (ARAs): (A) from the abdominal aorta (AA), (B) from the typical single renal artery (RA), (C) from other vessels, such as the common iliac artery (CIA) (C1), the superior mesenteric artery (SMA) (C2), and the celiac trunk (CT) (C3).

The abdominal aorta was the most common origin of ARAs (Figure 3), with a pooled prevalence of 88.39% (95% CI: 79.55%–95.25%). The typical RA followed, with an estimated pooled prevalence of 8.34% (95% CI: 1.77%–17.93%). Other rare origins (common iliac, celiac trunk, superior mesenteric artery) had a pooled prevalence of less than 0.01%.

**FIGURE 4:**
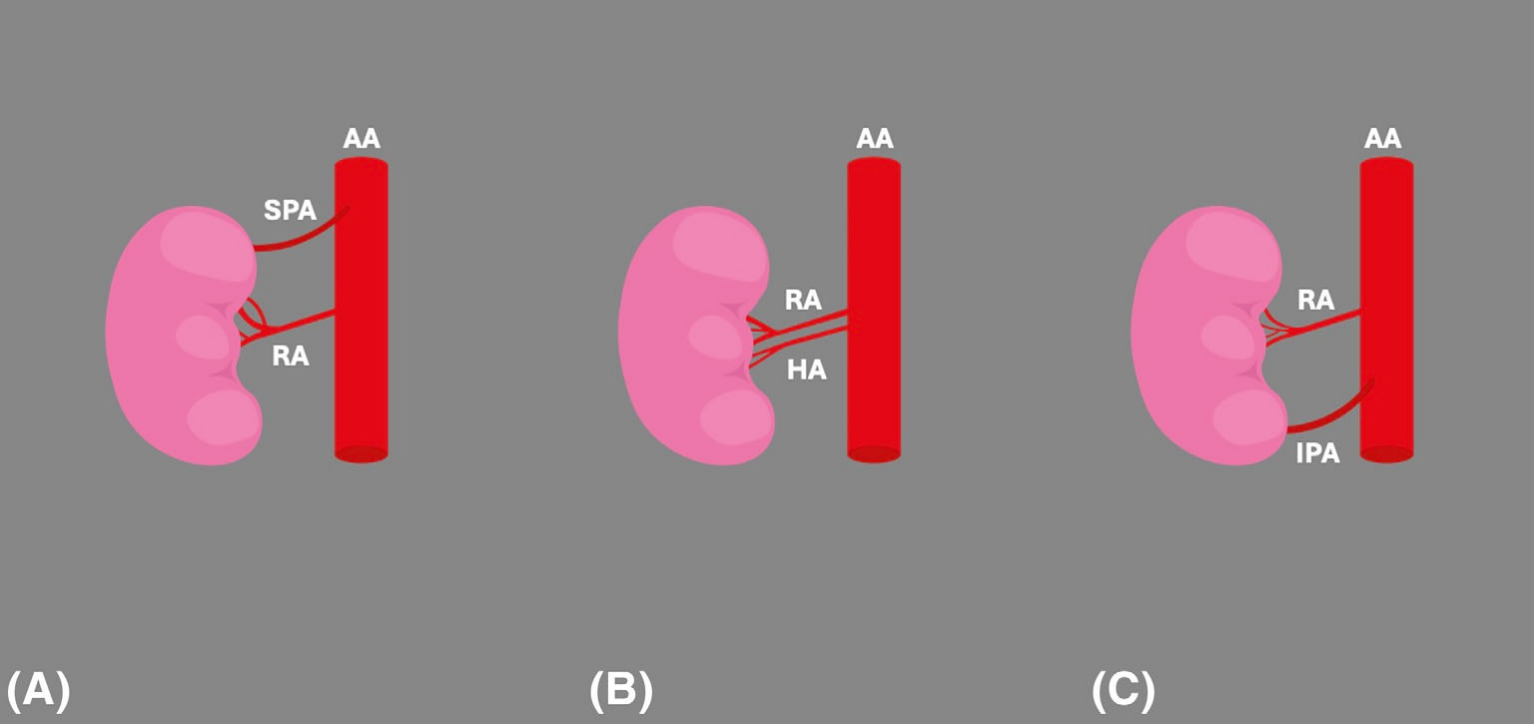
Terminations of the accessory renal arteries (ARAs): (A) superior polar (SPA), (B) hilar artery (HA), (C) inferior polar artery (IPA). AA, abdominal aorta; RA, renal artery.

The hilar artery was identified as the ARA termination in 8.83% (95% CI: 5.36%–13.02%), the superior polar in 8.02% (95% CI: 5.50%–10.96%), and the inferior polar in 6.69% (95% CI: 4.68%–9.02%) (Figure 4).

Eighty‐three (83) studies had a high risk of bias according to AQUA Tool analysis, and most of the meta‐analysis results had considerable heterogeneity. The typical RA pooled prevalence Doi plot retrieved an LFK index of −1 (no asymmetry), and the ARA pooled prevalence Doi plot retrieved an LFK index of +1.01 (minor asymmetry) (Data S1).

## 4 DISCUSSION

Applied anatomy is of paramount importance in clinical practice, especially for identifying vascular variants, which can complicate typical procedures. Findings of variants should be systematically reviewed with meta‐analysis (Henry et al., 2016; Koziej et al., 2022; Nikov et al., 2023; Ostrowski et al., 2023; Solewski et al., 2021; Triantafyllou et al., 2024; Whitley et al., 2021; Yurasakpong et al., 2022). The pooled prevalence of ARAs is reported in the present systematic review with meta‐analysis, including variant numbers, origins, and terminations of the arteries, highlighting symmetrical and asymmetrical morphologies.

The high morphological variability of RAs can be attributed to the complex embryological development of the kidneys. Kidney development begins in the sacral region, followed by ascent to the lumbar region and their final position, which is a key factor. During this process, the caudal branches of the lateral splanchnic vessels degenerate as more proximal vessels, closer to the kidneys’ final location, take their place. However, failure of regression can lead to the formation of ARAs between the 11th thoracic and 5th lumbar vertebral levels (Tubbs et al., 2016).

The prevalence of ARAs has been extensively studied for many decades. The literature search identified 111 eligible studies reporting their incidence. The anatomical‐clinical significance of this variation justifies the extensive research. We reviewed all these studies systematically, focusing initially on the presence of typical RAs or ARAs and their laterality (unilateral or bilateral) and side of appearance (right or left). We also investigated the origin and termination of the ARAs. We recommend that researchers use this exact method to study ARA variants clearly and systematically. Using the random‐effects model, we estimated the overall prevalence of ARAs to be 21.10%, the typical RA being present in 78.92% of the population. The current meta‐analysis did not reveal side differences for each ARA variant (Table 5). We found symmetrical RA anatomy (the same number of RAs bilaterally) in 74.50% of cases, and asymmetrical RA anatomy (a different number of RAs bilaterally) in 25.50%. Detailed overviews of the pooled prevalence of ARAs, including subgroup analyses based on geographical distribution, type of study, side, sex, and laterality, are provided in Tables 3, 4, and 5. Some individual studies have reported geographical, side, or sex predominance of ARAs (Gulas et al., 2016; Gulas et al., 2018), but our meta‐analysis showed no significant effects. Geographical difference was further evaluated by excluding the Oceania population, which had few studies (*k*=2), but no nationality influence could be discerned. This is not uncommon, because ethnic differences are reported in individual studies, while meta‐analysis is a reliable statistical method that summarizes several studies.

The most common ARA origin was the abdominal aorta, estimated at 88.39%, followed by an origin from the typical RA (8.34%). Other origins should be considered rare (<0.01%). According to *Bergman's Comprehensive Encyclopedia of Anatomic Variations*, the ARA has rarer origins from the thoracic aorta (Delasotta et al., 2015), the celiac trunk (Rusu & Manta, 2018), and the inferior phrenic (Li, Jie, et al., 2021; Li, Xia, et al., 2021), superior or inferior mesenteric (Balcerzak et al., 2022), common iliac and external or internal iliac arteries (Tubbs et al., 2016).

Gulas et al. (2016) highlighted that an ARA could be associated with testicular or suprarenal vessel variants. However, only case reports have described this coexistence (Kigata & Kobayashi, 2024). Variations in the RA and renal veins (RV) have also been reported to coexist. Hostiuc et al. (2019) estimated the pooled prevalence of accessory RVs at 16.7% in their meta‐analysis of RV variants.

The occurrence of ARAs is important for surgeons, radiologists, and clinicians, particularly in the context of kidney transplantation. There is an ongoing debate among surgeons regarding the pros and cons of using an ARA in kidney transplant procedures. A meta‐analysis by Zorgdrager et al. (2016) compared the outcomes of kidney transplants in patients with and without ARAs and showed that graft or patient survival in patients with ARAs did not differ significantly from those without. However, complication rates varied noticeably, particularly when the recipient had an ARA, which led to a higher incidence of vascular problems such as thrombosis and bleeding, especially in cases with severe atherosclerosis. Also, patients with ARAs were more likely to experience delayed graft function. The findings of this meta‐analysis indicate that an ARA is a negative factor in transplant surgery. During the operation, ARA anastomoses are typically performed end‐to‐side or end‐to‐end into the iliac vessels, arteries supplying less than 5%–10% of the kidney often being ligated. However, ligating the inferior polar type of ARA could lead to ureteral ischemia or necrosis (Zorgdrager et al., 2016). This could be attributable to the anterior course of the inferior polar arteries to the ureteropelvic junction, which could also cause hydronephrosis (Natsis et al., 2014).

In addition to surgical complications, an ARA has been linked to resistant hypertension. Percutaneous sympathetic denervation of the RA, a minimally invasive procedure that reduces sympathetic stimulation and blood pressure, has been proposed as a treatment option (Kasprzycki et al., 2023). However, some patients do not respond to it, and an ARA has been identified as a factor in this lack of response, prompting some randomized trials to exclude such patients (Kasprzycki et al., 2023). ARAs have also been associated with resistant hypertension owing to their suggested role in activating the renin‐angiotensin‐aldosterone system. The role of ARAs in transplantation and RA sympathetic denervation has been well established. However, their association with resistant hypertension is still debated (Zorgdrager et al., 2016; Tubbs et al., 2016; Kasprzycki et al., 2023). Techniques such as angiography, computed tomography, ultrasonography, and magnetic resonance imaging have been used to visualize the renal vasculature. The arterial phase of three‐dimensional computed tomography angiography is considered the gold standard for depicting an ARA (Gulas et al., 2016). Nevertheless, secondary hypertension due to stenosis of an ARA is rare, described in very few case reports (Chung & Millner, 2020). Lv et al. (2023) reported that the incidence of RA variations and an ARA were significantly higher in the affected kidney than the healthy kidney. Patients with an ARA in the affected kidney had a larger maximum tumor diameter, a higher Fuhrman grade, and more exophytic growth. An ARA in the affected kidney and the maximum tumor diameter are independent predictors of high‐grade renal cell carcinoma (Lv et al., 2023).

It is essential to acknowledge some limitations of the current systematic review with meta‐analysis. First, some earlier non‐English literature could be missing from our literature search. However, our sample (*k*=111) was large enough for a reliable meta‐analysis. Most of the studies (83) had a high risk of bias, and there was considerable heterogeneity in the meta‐analysis results. However, this is common among systematic reviews in anatomy (Henry et al., 2016; Henry et al., 2017). The meta‐analysis did not include all the literature needed to depict different patterns and subtypes of ARA accurately (Lippert & Pabst, 1988). Nevertheless, we presented the laterality of ARAs as symmetry or asymmetry. While most studies reported the number of bilateral ARAs (symmetry), they did not report the different combinations of ARA asymmetry (different numbers bilaterally).

## 5 CONCLUSIONS

We conducted a comprehensive review with meta‐analysis to examine the prevalence, origin, and termination of ARAs. Our findings revealed that the typical RA is present in 78.92% of the population, and ARAs had an overall prevalence of 21.10%. The most common RA variants included one ARA (18.67%) and two ARAs (1.80%). Instances of more than two ARAs appeared rare. The abdominal aorta was identified as the most common origin of the ARA (88.39%). A deep understanding of the morphological anatomy of ARAs is essential for anatomists, radiologists, and surgeons as such a vessel can complicate such procedures as treatment for resistant hypertension or kidney transplantation. Using three‐dimensional computed tomography angiography for visualization is vital for assessing related pathologies.

## Supporting information


**Data S1.** Supporting Information.
