# Supplementary material for: The accessory renal arteries: A systematic review with meta‐analysis
Source: Clin Anat. 2024 Dec 8;38(6):660–72. doi: 10.1002/ca.24255 (PMC12358822; doi:10.1002/ca.24255)
Supplement: Supplementary file 1 — Data S1. Supporting Information. [file CA-38-660-s001.docx]

**Supporting Information**

Statistical figures from our meta-analysis using the R programming software.


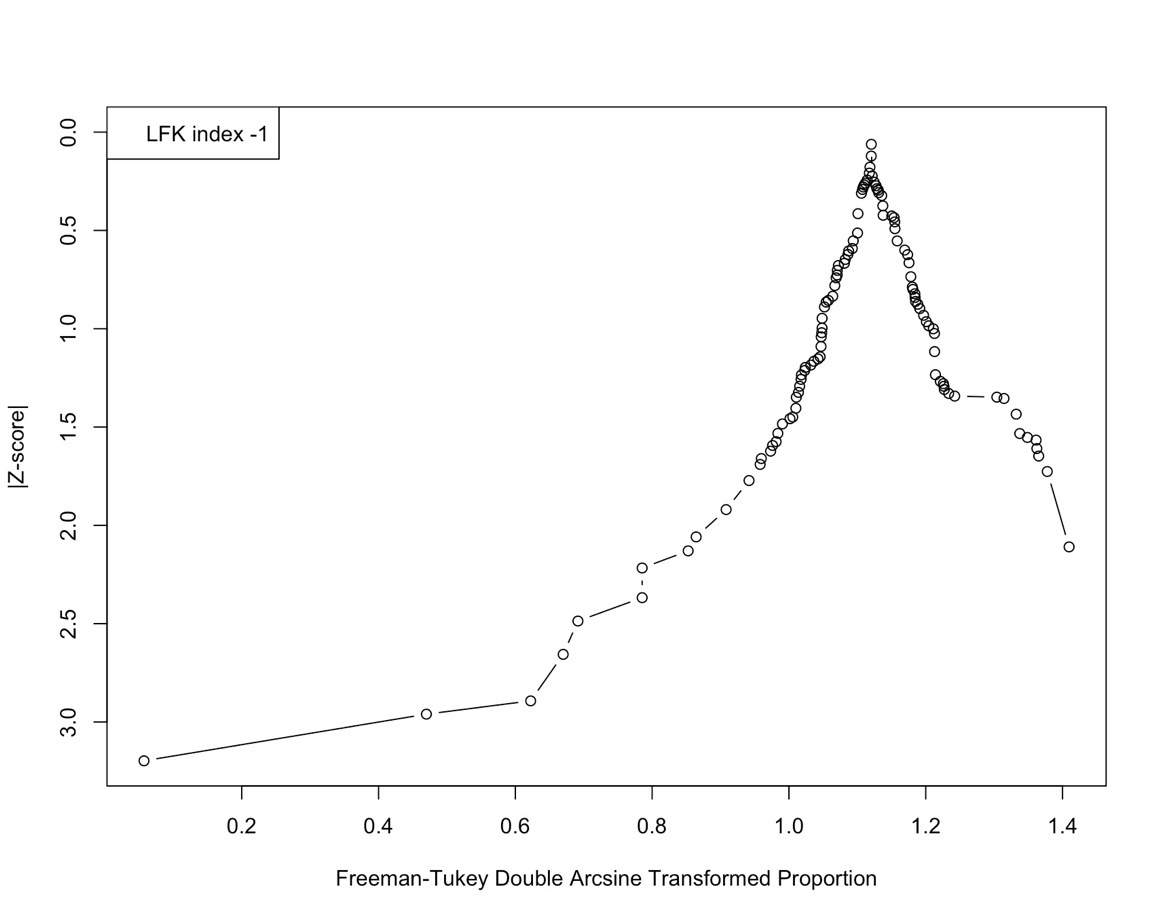


**Figure 1**. DOI plot with LFK index for typical RA pooled prevalence.


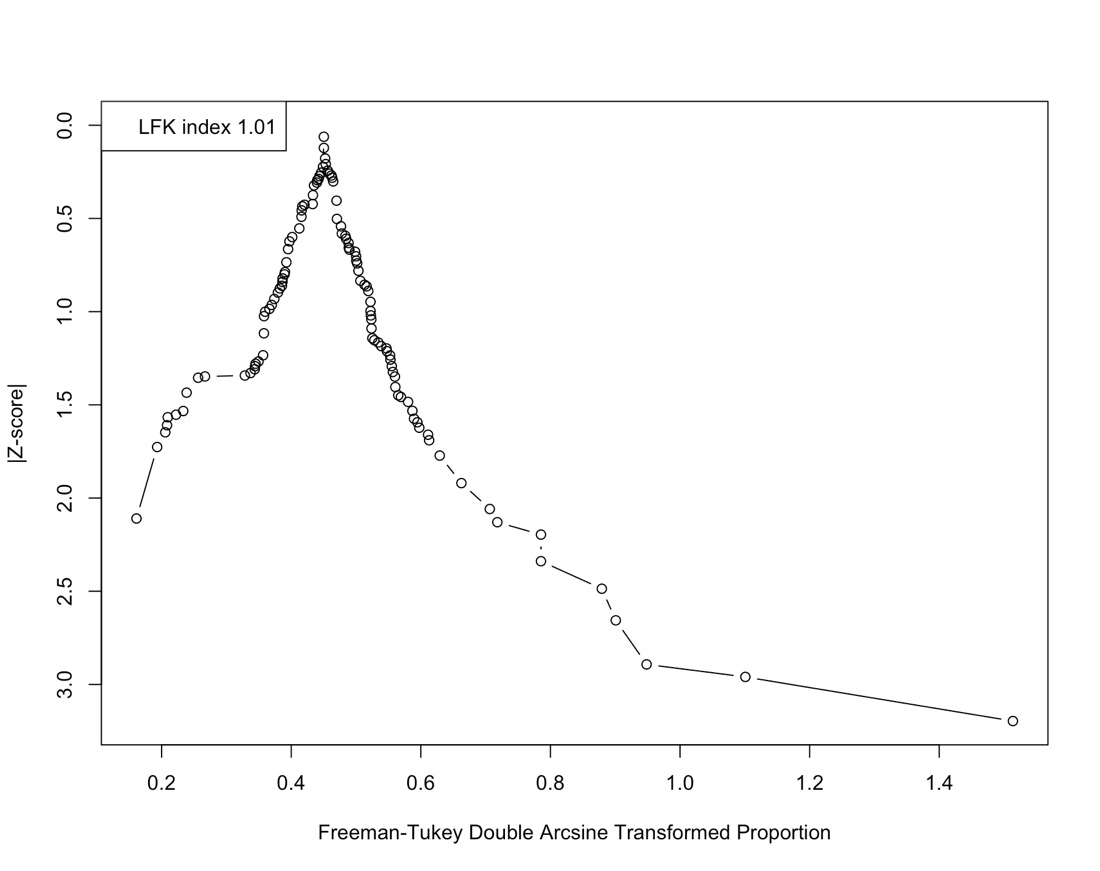


**Figure 2.** DOI plot with LFK index for ARA pooled prevalence.
